# Supplementary material for: Cloud BioLinux: pre-configured and on-demand bioinformatics computing for the genomics community
Source: BMC Bioinformatics. 2012 Mar 19;13:42. doi: 10.1186/1471-2105-13-42 (PMC3372431; doi:10.1186/1471-2105-13-42)
Supplement: Additional file 1 — Supplementary 1 Cloud BioLinux software documentation in the form of a mini, self-contained website. Users need to download and uncompress the .zip file, and open through a web browser the "index.html" file available on the main directory. (ZIP 1823 kb). [file 1471-2105-13-42-S1.ZIP › Cloud-BioLinux-Package-Documentation/docs/ape.html]

Bio-Linux Software Documentation Pages

Back to search form

## ape

|  |  |
| --- | --- |
| Name | ape |
| Description | **Ape** provides functions for reading, writing, plotting, and manipulating phylogenetic trees, analyses of comparative data in a phylogenetic framework, analyses of diversification and macroevolution, computing distances from allelic and nucleotide data, reading nucleotide sequences, and several tools such as Mantel's test, computation of minimum spanning tree, or the population parameter theta based on various approaches. The package is installed in the "/usr/software/ape/ape/R" directory  **References:**  Emmanuel Paradis is the maintainer for this package, and her homepage can be found here  A PDF Manual for Ape can be found here |
| Homepage | http://ape.mpl.ird.fr/ |
| Remote Documentation | http://bioinformatics.oxfordjournals.org/cgi/reprint/20/2/289   http://ape.mpl.ird.fr/ape\_features.html   http://ape.mpl.ird.fr/a |
